# Novel urine-based DNA methylation biomarkers for urothelial bladder carcinoma detection in patients with hematuria

**DOI:** 10.1080/2090598X.2023.2208492

**Published:** 2023-05-14

**Authors:** Hassan F. Abol-Elnazer, Amira Awadalla, Asmaa E. Ahmed, Hassan Abol-Enein, Munir Ali Al Ganzouri, Amr A. Elsawy

**Affiliations:** a Genetics Unit, Mansoura University Hospital, Mansoura University, Mansoura, Egypt; b Center of Excellent for Genome and Cancer Research (CEG-CR), Urology and Nephrology Center, Mansoura University, Mansoura, Egypt; c Urology department, Urology and Nephrology Center, Mansoura University, Mansoura, Egypt; d Faculty of Science, Ain Shams University, Cairo, Egypt

**Keywords:** Urothelial bladder carcinoma, hematuria, biomarkers, DNA methylation, cystoscopy

## Abstract

**Background:**

Urothelial bladder carcinoma (UBC) is usually detected during work-up for hematuria. Cystoscopy and/or contrast-enhanced imaging are the gold standard tools for UBC diagnosis, despite limited by being invasive, expensive and low yield in small flat tumors.

**Objectives:**

To assess the diagnostic performance of urine-based DNA methylation of six genes (GATA4, P16, P14, APC, CDH1 and CD99) for UBC detection in patients with hematuria.

**Patients and methods:**

Voided urine was collected from consecutive patients presented with hematuria for urine cytology and DNA methylation assay of the assigned genes using methylation-specific Polymerase Chain Reaction (PCR). Further assessment by office cystoscopy and imaging with subsequent inpatient cystoscopic biopsy for positive findings was done. The diagnostic characteristics of DNA methylation and urine cytology were assessed based on its capability to predict UBC.

**Results:**

We included 246 patients in the study with identified macroscopic hematuria in 204 (82.9%) patients. Positive cytology was found in 78 (31.7%) patients. DNA methylation of GATA4, P16, P14, APC, CDH1 and CD99 genes was identified in 127 (51.6%), 52 (21.1%), 117 (47.6%), 106 (43.1%), 90 (36.6%) and 71 (28.9%) patients, respectively. The sensitivity of the assigned genes for UBC detection ranges from 35% (95%CI: 31–39) to 83% (95%CI: 79–87). Optimal specificity (SP) (100%) was noted for P16, APC and CDH1 genes. While for the other genes (GATA4, P14 and CD99), the SP was 95% (95%CI: 92–98), 96% (95%CI: 92–99) and 97% (95%CI: 93–99), respectively. On multivariate logistic regression analysis, all genes exclusively demonstrated independent prediction of UBC. On receiver operator characteristic (ROC) analysis, all tested genes methylation showed superior area under the curve (AUC) when compared to urine cytology.

**Conclusions:**

We have developed a novel urine-based DNA methylation assay for detection of UBC in patients with hematuria with superior diagnostic performance and independent predictive capacity over urine cytology.

## Introduction

According to GLOBOCAN data, bladder cancer (BC) is considered a major health problem that represents 3% of all cancer diagnoses [1]. Urothelial bladder carcinoma (UBC) accounts for the vast majority (>90%) of BC cases with predominance of non-muscle invasive disease (Ta, Tis or T1) in 75% of patients, while others show muscle invasion (T2–4) [1].

In refereed population, UBC is usually diagnosed as a result of work-up for hematuria at a rate of 2–5% following an evaluation of asymptomatic microscopic hematuria [2], and, up to 5–15% of patients with macroscopic hematuria [3]. Therefore, a timely prompt evaluation of hematuria can give to earlier diagnosis and better survival of UBC [4].

Currently, cystoscopy/cross sectional imaging is the gold standard tools for UBC diagnosis in patients with hematuria [5]. Unfortunately, these costly, invasive diagnostics could miss early, small/flat bladder lesions [6]. Urine cytology has been proposed as a non-invasive alternative test with high specificity; however, it lacks sensitivity for diagnosis of low grade (LG) tumors [7].

Over the last decades, multiple researches have output different markers for UBC diagnosis. Based on their target of assessment, these markers include screening for soluble antigens (BTA-Stat, NMP-22, surviving, etc.), cell surface antigens (Cytokeratins and UroVysion), genomic markers (Cxbladder and Xpert) and urinary metabolomics (*CRAT and SLC25A20*) [8]. However, most of these markers are limited by unsatisfying diagnostic performance, high cost or lack of validation [7].

Several preliminary studies have shown that DNA methylation, a critical step in transcription regulation, is chemically stable and can be precisely quantified, making it an attractive marker for UBC detection [9]. Both local and global DNA methylations in BC specimens are usually associated with inactivation of tumor suppressor genes. These methylation changes could be effectively identified in urine sediments as well as tumor tissues [10].

In the current literature, multiple studies investigated the performance of DNA methylation on either individual or panel genes with reported sensitivity (SN) and specificity (SP) values that range from 40% to 95% and 10% to 100%, respectively [9]. Most of these studies were limited by tumor characteristic heterogeneity (majority were ≥T2 and high grade (HG) disease) which did not reflect the daily practice, inclusion of different BC histological variants, lack of external validation and small sample size [11].

The aim of our study is to assess the diagnostic performance of novel urine-based DNA methylation of six genes (*GATA4, P16, P14, APC, CDH1 and CD99*) for UBC detection in patients with hematuria. Moreover, we investigated the methylation pattern of these genes in different stages and grades of UBC.

## Study design, settings and participants

### Study participants

After Institutional Review Board approval, patients presented with macroscopic or microscopic hematuria were evaluated for eligibility to our study. Eligible patients were asked to participate in this study and were enrolled after signing an informed consent form.

### Inclusion and exclusion criteria

Inclusion criteria included patients with hematuria (macroscopic or microscopic). On the other hand, patients who had history of BC, pelvic irradiation, bleeding diathesis or receiving anticoagulants and patients with upper urinary tract neoplasm or urolithiasis detected by cross-sectional imaging were excluded.

### Voided urine cytology, urine-based DNA methylation evaluation

#### Urine cytology evaluation

Prior to outpatient check cystoscopy, a voided urine sample was collected and assessed according to the Paris classification system [12]. Positive results were defined when suspicious or malignant results are obtained, while hyperplastic and negative samples were defined negative for malignancy.

#### Urine methylation tests

Voided urine samples were obtained in a sterile fashion (40 ml per each patient). Collected samples were frozen to −25^◦^C within 60 min of collection. In a central laboratory, genomic DNA was isolated from the collected samples and analyzed using methylation-specific Polymerase Chain Reaction (PCR). The operators at the central laboratory were blinded to the cytology/cystoscopy results.

DNA isolation was done using the standard method (Geneture, Luoyang, China). After that, quantification of the samples was done using DNA Quantitation PicoGreen Kit (Thermofisher, Massachusetts, US). As directed by the manufacturer, 1.5 mg of DNA was utilized for bisulphite stabilization using the available kit (OptuIN, Ca, USA). In this reaction, unmethylated cytosine filtrates are deaminated resulting in its conversion to uracil, while methylated residues are kept unmodified. The analyte quantifications of the modified DNA were processed using real-time assays which included parallel amplification and quantification cycles using specific probe and primer (***Supplementary table***) for each analyte.

The methylation results were defined using special generation software (Applied Biosystems), which is expressed as cycle threshold values. Cell lines with identified methylation condition for each tested gene were applied in each cycle as positive and negative status and included in the process at the extraction phase.

Validity of urine sample was ensured when at least ten copies of *ACTB* gene (***Supplementary table***) were identified in the isolated DNA from the urine samples. Only valid samples were assessed for methylation status of the tested six genes.

The results were expressed as gene copy numbers. Methylated urine sample for each gene was defined when at least ten copies of this gene were measured. Unmethylated samples were defined when <10 copies of assigned gene were measured. These cut-off values were calculated during the performance analysis of the assigned gene sets.

### Upper tract assessment and office cystoscopy procedures

Within 2 weeks of urine sample collections, study participants were thoroughly evaluated by cross-sectional imaging and outpatient check cystoscopy.

Cross-sectional imaging in the form of multiphasic computed tomography was done to exclude upper urinary tract malignancies or urinary stones (unless contraindicated; magnetic resonance imaging was done).

All office cystoscopy procedures were done using flexible white light cystoscopy. Precise scanning of the bladder was done. According to the findings obtained, patients were categorized into three groups; (1) cystoscopy-positive (gross bladder lesion/s), (2) cystoscopy-suspicious (hyperemic/suspicious mucosa) and (3) cystoscopy-free (no gross lesions). For cystoscopy-positive and suspicious patients, transurethral resection bladder biopsy was done within 2 weeks from the check cystoscopy.

Cystoscopy-free patients were followed-up accordingly (by either urgent clinic visit for patients with recurring hematuria or telephone call every 12 weeks for asymptomatic cases). On the other hand, patients with recurring hematuria and persistent/aggravated bladder symptoms were assigned to re-check cystoscopy and cytology. Positive/suspicious re-cystoscopy (and/or cytology) was evaluated by biopsy to confirm or exclude malignancy.

### Outcome measures

The primary outcome is to assess the diagnostic performance of urine cytology and evaluated genes methylation (*GATA4, P16, P14, APC, CDH1 and CD99)* for UBC diagnosis in patients with hematuria. Subsequently, SN, SP, positive and negative predictive values (PPV, NPV) were calculated. In addition, further capability of these genes hypermethylation for prediction of different stages and grades of UBC was assessed.

### Statistical analysis

We computed all data using IBM statistical software v. 19. Chi-square and Fisher exact tests were utilized to assess the association between categorical variables, while for the comparison of urine cytology and genes hypermethylation as regard to cystoscopy/biopsy, McNemar test was utilized. Logistic regression analysis was utilized for multivariate analysis of significant predictors of UBC in study participants. A receiver operator characteristic (ROC) curve was constructed for this logistic regression by plotting the true positive finding (SN) against the false-positive findings (100-SP). The area under the curve (AUC) was estimated. Boxplots were utilized to demonstrate the genes methylation copies and its predictive capacity for UBC stage and grade. A critical two-sided P-value <0.05 was used for statistically significant differences.

## Results

### Base line demographics

**Figure 1.**
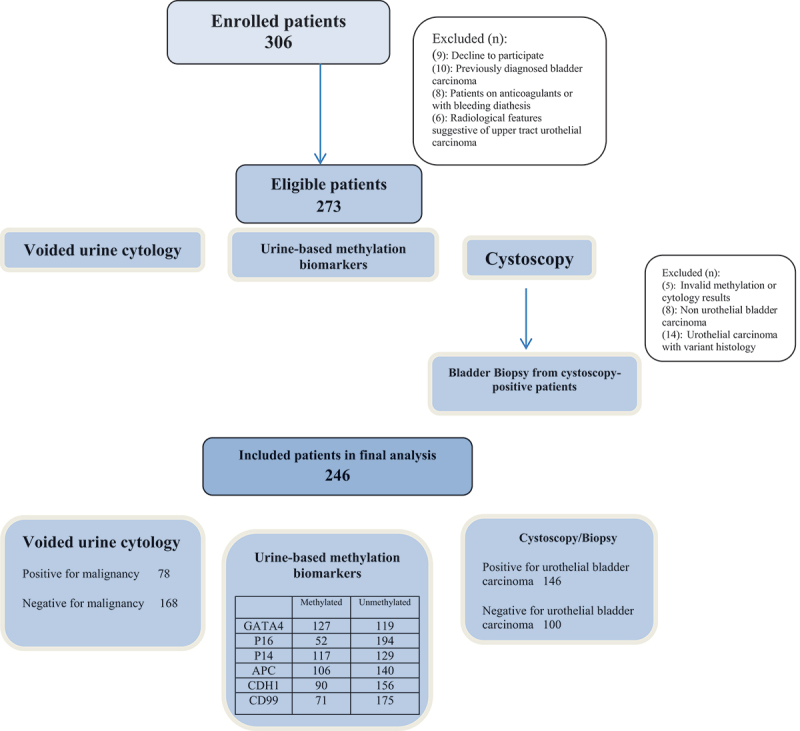
Study flowchart.

From February 2019 and August 2021, 306 enrolled patients were assessed for study eligibility. After exclusion of patients with invalid methylation or cytology results, non-UBC and variant histology UBC, 246 patients (201 males, 45 females) with a median (range) age of 58 (34–99) years were included in the final analysis. The study flow chart is shown in Figure (1) while baseline demographics of the study participants are illustrated in Table (1).

**Table 1.** Patients’ demographics.

Median age in years (range)	58 (34-99)
Sex *n* (%)	
• Male	201 (81.7)
• Female	45 (18.3)
Hematuria type *n* (%)	
•Macroscopic	204 (82.9)
•Microscopic	42 (17.1)
Diabetes mellitus *n* (%)	
•No	184 (74.8)
•Yes	62 (25.2)
Hypertension *n* (%)	
•No	174 (70.7)
•Yes	72 (29.3)
Concomitant dysuria/suprapubic pain/urgency *n* (%)	
•No	184 (74.8)
•Yes	62 (25.2)
History of urological problems *n* (%)	
•No	194 (78.9)
•Yes	52 (21.1)
○ Urolithiasis	16 (6.5)
○ Bladder outlet obstruction	16 (6.5)
○ Urinary tract infections	20 (8.1)
Concomitant indwelling bladder foreign body *n* (%)	
•No	238 (96.7)
•Yes	8 (3.3)
○ Catheter	3 (1.3)
○ Bladder stone	5 (2)
Smoking history *n* (%)	
•Never	90 (36.6)
•Former	75 (30.5)
•Current	81(32.9)
Cytology result *n* (%)	
•Negative	168 (68.3)
•Positive	78 (31.7)
Cystoscopy findings *n* (%)	
•Free	100 (40.7)
•bladder lesion	146 (59.3)
Urine methylation assay target *n* (%)	
•GATA4	
Methylated	127 (51.6)
Unmethylated	119 (48.4)
•P16	
Methylated	52 (21.1)
Unmethylated	194 (78.9)
•P14	
Methylated	117 (47.6)
Unmethylated	129 (52.4)
•APC	
Methylated	106 (43.1)
Unmethylated	140 (56.9)
•CDH1	
Methylated	90 (36.6)
Unmethylated	156 (63.4)
•CD99	
Methylated	71 (28.9)
Unmethylated	175 (71.1)
Biopsy findings in positive cystoscopy (Urothelial carcinoma) *n* (%)	
Stage (TNM)	
○ Ta, Tis, T1	31 (12.6)
○ ≥ T2	115 (46.7)
Grade (WHO/ISUP 2004)	
○ Low grade	7 (2.8)
○ High grade	139 (56.5)

### Voided urine cytology and DNA methylation assay

As demonstrated in Table (1), negative and positive cytology were found in 168 (68.3%) and 78 (31.7%) patients, respectively. DNA hypermethylation of *GATA4, P16, P14, APC, CDH1 and CD99 genes* was identified in 127 (51.6%), 52 (21.1%), 117 (47.6%), 106 (43.1%), 90 (36.6%) and 71 (28.9%) patients, respectively. On the other hand, cystoscopy/biopsy showed negative and positive findings for UBC in 100 (40.7%) and 146 (59.3%) patients, respectively.

### Outcome measures

*The diag*nostic performance characteristics of urine cytology and evaluated genes DNA methylation are clarified in Table (2). The SN of the assigned genes for UBC detection ranges from 35% (95%CI: 31–39) to 83% (95%CI: 79–87) for *P16* and *GATA4*, respectively. On the other hand, optimal SP (100%) was noted for *P16, APC and CDH1* genes. While for the other genes (*GATA4, P14 and CD99*), the SP was 95% (95%CI: 92–98), 96% (95%CI: 92–99) and 97% (95%CI: 93–99), respectively.

**Table 2.** Cross tables of different urine assay methylation targets and urine cytology in comparison to cystoscopy/biopsy results in study participants.

	Urothelial bladder carcinoma by cystoscopy and biopsy n %		Total	Diagnostic characteristics % (95%CI)
	Positive	Negative		
GATA4 Methylated Unmethylated	122 (83.6%) 5 (5%)	24 (16.4%) 95 (95%)	127 119	SN 83.6% (95%CI: 79–87) SP 95% (95%CI: 92–98) PPV 96.1% (95%CI: 92–99) NPV 79.8% (95%CI: 75–84)
Total *Count*	146	100	246	
P16 Methylated Unmethylated	52 (35.6%) 94 (64.4%)	0 100 (100%)	52 194	SN 35.6% (95%CI: 31–39) SP 100% PPV 100% NPV 51.5% (95%CI: 46–55)
Total *Count*	146	100	246	
P14 Methylated Unmethylated	113 (77.4%) 96 (96%)	4 (4%) 96 (96%)	117 129	SN 77.4% (95%CI: 72–83) SP 96% (95%CI: 92–99) PPV 96.6% (95%CI: 93–99) NPV 74.4% (95%CI: 71–78)
Total *Count*	146	100	246	
APC Methylated Unmethylated	106 (72.6%) 40 (27.4%)	0 100 (100%)	106 140	SN 72.6% (95%CI: 67–79) SP 100% PPV 100% NPV 71.4% (95%CI: 66–75)
Total *Count*	146	100	246	
CDH1 Methylated Unmethylated	90 (61.6%) 56 (38.4%)	0 100 (100%)	90 156	SN 61.6% (95%CI: 57–66) SP 100% PPV 100% NPV 64.1% (95%CI: 60–68)
Total *Count*	146	100	246	
CD99 Methylated Unmethylated	68 (46.6%) 78 (53.4%)	3 (3%) 97 (97%)	71 175	SN 46.6% (95%CI: 42–50) SP 97% (95%CI: 93–99) PPV 95.8% (95%CI: 91–99) NPV 55.4% (95%CI: 51–59)
Total *Count*	146	100	246	
Urine cytology Positive *Count* Negative *Count*	57 (39%) 89 (61%)	21 (21%) 79 (79%)	78 168	SN 39% (95%CI: 36–42) SP 79% (95%CI: 76–84) PPV 84.5% (95%CI: 81–88) NPV 61% (95%CI: 57–65)
Total *Count*	146	100	246	

Note: SN: Sensitivity, SP: specificity, PPV: Positive predictive value, NPV: Negative predictive value.

Notably, the evaluated genes methylation showed superiority to voided urine cytology as regard to SN (apart from *P16*), SP (all six genes), PPV (all six genes) and NPV (apart from *p16* and *CD99*) for UBC detection (McNemar test; *p* < 0.001) Table (2).

**Figure 2.**
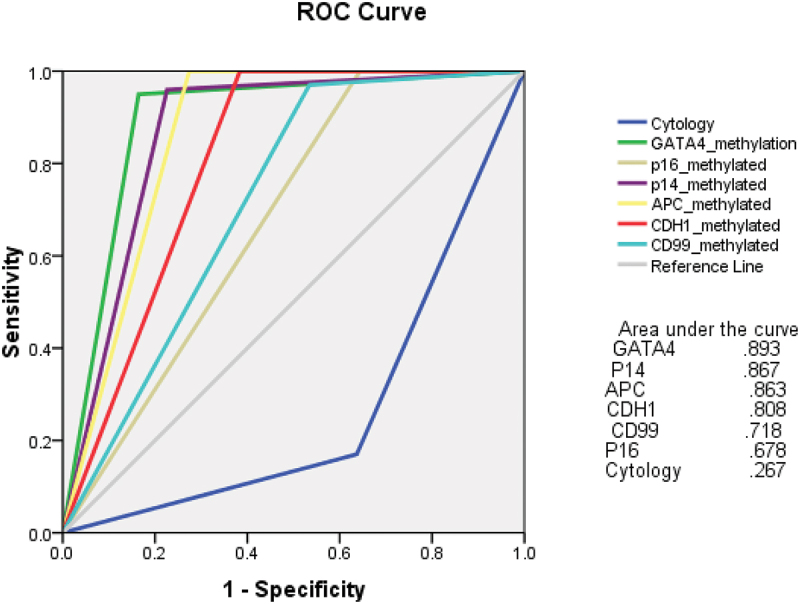
Receiver operator characteristic (ROC) curve for urine-based methylation biomarkers and urine cytology for urothelial bladder carcinoma detection in the study cohort.

Table (3) shows the univariate and multivariate logistic regression analysis of the independent predictors of UBC in the study participants. All the six genes demonstrated exclusively significant findings for prediction of UBC (*GATA4*: HR 4.2 (95%CI 2.78–8.24) *p* = 0.001, *P16*: HR 2.1 (95%CI 1.72–3.12) *p* = 0.003, *P14*: HR 1.4 (95%CI 1.1–1.8) *p* = 0.007, *APC*: HR 2.1 (95%CI 1.8–2.5) *p* = 0.006, *CDH1*: HR 2.32 (95%CI 1.92–2.73) *p* = 0.002, *CD99*: HR 2.12 (1.83–2.53) *p* = 0.003). On ROC analysis, DNA methylation of all the study genes showed superior AUC when compared to urine cytology (Figure 2).

**Table 3.** Univariate and multivariate analyses for predictors of positive cystoscopy/biopsy for urothelial bladder carcinoma (UBC).

Patient and tumor characteristics	UBC by cystoscopy and biopsy NoYes N (%) N (%)	Univariate analysis HR (95%CI) p value	Multivariate analysis HR (95%CI) p value
Median age in years (Range)	56 (36–75) 60 (34–99)	1.01 (0.9–1.03) 0.47	
Sex *n* (%) Male Female	80 (39.8) 121 (60.2) 20 (44.4) 25 (55.6)	1.2 (0.9–1.5) 0.56	
Hematuria *n* (%) Macroscopic Microscopic	82 (40.2) 122 (59.8) 18 (42.9) 24 (57.1)	1.4 (0.95–1.85) 0.75	
Concomitant dysuria/suprapubic pain/urgency *n* (%) No Yes	73 (39.7) 111 (60.3) 27 (43.5) 35 (56.5)	1.2 (0.89–1.5) 0.59	
History of urological problems *n* (%) No Yes	81 (41.8) 113 (58.2) 19 (36.5) 33(63.5)	1.12 (0.8–1.4) 0.5	
Indwelling bladder foreign body *n* (%) No Yes	99 (41.6) 139 (58.4) 1 (12.5) 7 (87.5)	1.15 (0.88–1.34) 0.1	
Smoking *n* (%) Never Former Current	43 (47.8) 47 (52.2) 25 (33.3) 50 (66.7) 32 (39.5) 49 (60.5)	0.92 (0.87–1.23) 0.17	
Cytology result *n* (%) Negative Positive	83 (61) 53 (39) 17 (15.5) 93 (84.5)	1.17 (0.74–0.1.64) 0.07	
Urine methylation assay target n (%) GATA4 Methylated Unmethylated P16 Methylated Unmethylated P14 Methylated Unmethylated APC Methylated Unmethylated CDH1 Methylated Unmethylated CD99 Methylated Unmethylated	5 (3.9) 122 (96.1) 95 (79.8) 24 (20.2) 0 52 (100) 100 (51.5) 94 (48.5) 4 (3.4) 113 (96.6) 96 (74.4) 33 (25.6) 0 106 (100) 100 (71.4) 40 (28.6) 0 90 (100) 100 (64.1) 56 (35.9) 3 (4.2) 68 (95.8) 97(55.4) 78 (44.6)	**2.58 (1.94–2.94) <0.001** **3.42 (2.9–3.8) <0.001** **2.72 (2.34–2.98) <0.001** **4.9 (4.3–5.42) <0.001** **3.35 (2.97–3.86) <0.001** **2.67 (2.32–2.91) <0.001**	**4.2 (2.78–8.24) 0.001** **2.1 (1.72–3.12) 0.003** **1.4 (1.1–1.8) 0.007** **2.1 (1.8–2.5) 0.006** **2.32 (1.92–2.73) 0.002** **2.12 (1.83–2.53) 0.003**

**Figure 3.**
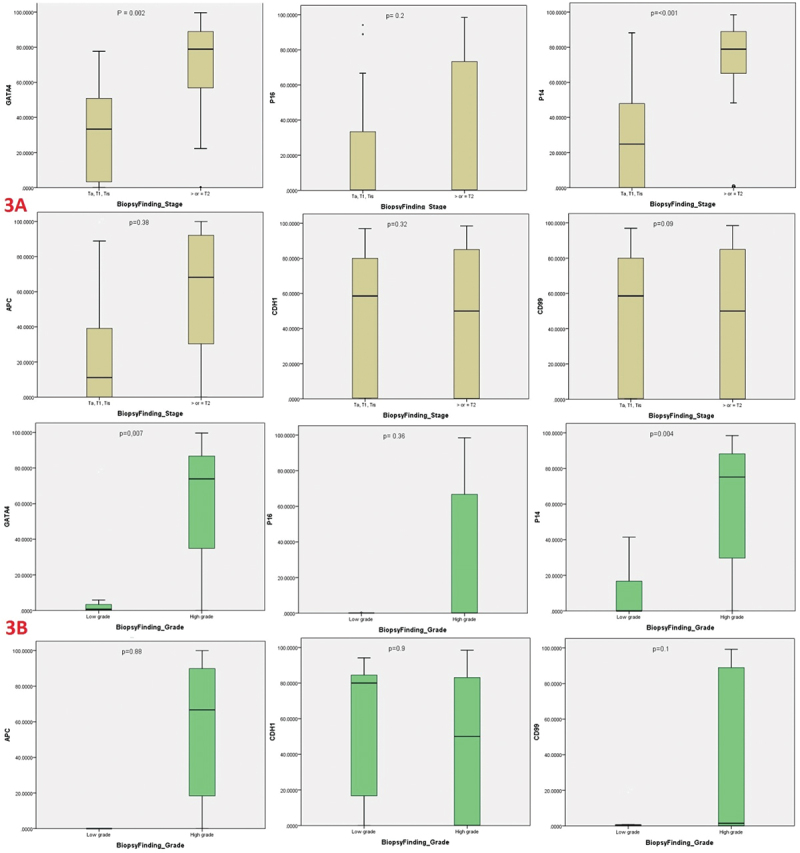
Boxplots of urine-based DNA methylation biomarkers GATA4, P16, P14, APC, CDH1 and CD99 for detection of different urothelial bladder carcinoma stages (3A) and grade (3B).

On sub-analysis of UBC cases, ≥T2 stage was identified in 115 patients, while HG tumors were observed in 139 patients. As illustrated in Figure 3, *GATA4* and *P14* genes methylation were significantly associated with ≥T2 stage disease (*p* = 0.002 and <0.001, respectively) and HG disease (*p* = 0.007 and 0.004, respectively).

The cystoscopy-free patients were followed-up for a median (range) period of 18 (7–36) months according to the predetermined protocol. Out of 100 patients, who completed the follow-up, persistent hematuria or aggravated bladder problems were reported in 12 patients. Re-cystoscopy and cytology were repeated in those patients, with evidence of UBC in one patient. Notably, DNA methylation was identified in the all six evaluated genes in this patient.

## Discussion

Hematuria is a significant clinical presentation that can imply an underlying serious pathology, especially in patients with macroscopic hematuria [13]. The recommendations of most available guidelines for the diagnostic work-up in those patients include invasive tests as diagnostic cystoscopy and/or contrast-enhanced imaging despite its limitations, namely high cost, substantial patient burden (pain) and health-care resources, associated adverse events and waiting schedules somewhere [14,15].

Reliable non-invasive markers might provide a helpful tool for clinicians to overcome the drawbacks of cystoscopies/imaging and prioritize patients for invasive diagnostic interventions [16]. Urine cytology was one of the early utilized to complement cystoscopy for detection of exfoliated tumor cells in urine despite its limitations, low sensitivity in LG tumors and inter-observer variability [17].

Thereafter, multiple urinary markers with different targets and mechanisms have been proposed and assessed over the last years [16]. One of the recent common markers to be introduced in this aspect is the study of some urine-based DNA methylation genes. Methylation of some genes can silence corresponding tumor suppressor gene/s [18].

The available literature on the clinical performance of DNA methylation in urine of UBC patients showed high variability with ranging SN between 50% and 95% and SP between 20% and 100% [9]. The explanation of this variation is essentially attributed to the heterogeneity of the evaluated gene/s, included heterogeneous patients with different histopathological variants, stage and grades, as well as different presentation patterns (primary vs. recurrent BC) [19].

In our study, we primarily included patients with hematuria (macroscopic or microscopic) with exclusion of patients with BC history. Most of the previous studies included heterogeneous patients (primary/recurrent/surveillance cases vs. healthy/symptomatic controls) [9]. Moreover, as compared to the previously published reports on DNA methylation markers for primary BC patients [20–22], our study included the largest cohort (146 BC and 100 symptomatic controls).

Among the evaluated six genes, our study assessed DNA methylation of two novel genes (*GATA4* and *CD99*) that were not previously investigated in the literature for this purpose. On the contrary, there is a paucity of the literature as regard to three genes (*CDH1, P14* and *P16*) with one report for each gene [23–25], these reports were limited by its small sample size (22–57 patients) and heterogeneous presentation.

Our findings showed varying SN of evaluated markers ranging from 35% (*P16*) to 83.5% (*GATA4*). As compared to SN of the previously investigated genes (*APC, CDH1, P14* and *P16)*, our findings were notably lower due to the nature of our study participants with exclusion of patients under BC surveillance and lesser patients with ≥T2/HG disease as these tumors probably harbor more molecular alterations [26].

On the other hand, the SP of the evaluated genes varies between 95% and 100%. The initial reports in the literature about single-gene DNA methylation showed compromised SP (0–60%), however, the nest reports which included gene panels rather than a single gene [9]. Our higher SP results could be attributed to the study design which included hematuria patients rather than healthy controls and inclusion of UBC with exclusion of other variant histologies [27].

When compared to urine cytology, superior results of our study genes were obtained as regard to independent diagnostic capacity in multivariate logistic regression analysis, as well as, AUC on ROC curve analysis.

In addition, *GATA4* and *P14* genes methylation were significantly associated with ≥T2 stage disease (*p* = 0.002 and <0.001, respectively) and HG disease (*p* = 0.007 and 0.004, respectively). This finding should add another diagnostic benefit of DNA methylation not only for UBC diagnosis but also for tumor characterization (stage/grade).

Our study is advantaged by its prospective nature, inclusion of consecutive hematuria patients with no BC history (better reflection of target population in daily practice than healthy controls), use of predefined threshold level of gene methylation, exclusion of non-UBC or UBC with abnormal variants and study of diagnostic capacity of evaluated genes for tumor stage and grade. On the other hand, there are some limitations in our study. First, our results need external validation in a larger cohort multicenter study. Second, our analysis included only hypermethylation study with lack of other analyses that can optimize the diagnostic capacity as mutation analysis [28]. Third, lack of further study of evaluated genes DNA methylation on future tumor recurrence and/or progression is a considerable limitation.

## Conclusions

We have developed a novel urine-based DNA methylation assay for non-invasive detection of UBC in patients with hematuria. Superior diagnostic performance and independent predictive capacity of the evaluated genes methylation over urine cytology are promising findings. Further longitudinal follow-up and external validation studies of our results are warranted before future implementation in clinical practice.

## Clinical trials registration

clinicaltrials.gov ID: NCT04714345

## Descriptive key message

The current study aimed to assess the diagnostic performance of novel urine-based DNA methylation of six genes (*GATA4, P16, P14, APC, CDH1 and CD99*) for UBC detection in patients with hematuria.

## List of abbreviations

AUC: Area under the curve
BC: Bladder cancer
HG: High-grade
LG: Low-grade
NPV: Negative predictive value
PCR: Polymerase chain reaction
PPV: Positive predictive value
ROCs: Receiver operator characteristics
SN: Sensitivity
SP: Specificity
UBC: Urothelial bladder carcinoma
